# Description and phylogeny of *Namalycastis jaya* sp. n. (Polychaeta, Nereididae, Namanereidinae) from the southwest coast of India

**DOI:** 10.3897/zookeys.238.4014

**Published:** 2012-11-05

**Authors:** Mathan Magesh, Sebastian Kvist, Christopher J. Glasby

**Affiliations:** 1 Department of Aquatic Biology and Fisheries, University of Kerala, Thiruvananthapuram, India, 695581; 2 Richard Gilder Graduate School, American Museum of Natural History, Central Park West at 79th Street, New York, NY 10024, USA; 3 Present address: Museum of Comparative Zoology, Department of Organismic and Evolutionary Biology, Harvard University, 26 Oxford Street, Cambridge, MA 02138, USA; 4 Museum and Art Gallery of the Northern Territory, GPO Box 4646, Darwin NT 0801, Australia

**Keywords:** Annelida, Polychaeta, Nereididae, *Namalycastis*, 16s rRNA, 18s rRNA, COI, phylogeny, systematics, new species, India

## Abstract

*Namalycastis jaya* **sp. n.** (Polychaeta: Nereididae: Namanereidinae)is described from the southern coast of Kerala in southwest India. One important characteristic feature of the species is the lack of notochaetae in all parapodia, a characteristic that it shares with at least two other species, *Namalycastis elobeyensis* Glasby, 1999and *Namalycastis hawaiiensis* Johnson, 1903. It differs from *Namalycastis elobeyensis* by virtue of its smaller antennae, unequal eye size, bilobed acicular neuropodial ligule and multi-incised pygidium rim. Moreover, it differs from *Namalycastis hawaiiensis* by having fewer teeth on the serrated blades of the sub-neuroacicular falciger in chaetiger 10, and by possessing finely serrated falcigers in posterior segments. Beyond morphological analyses, molecular phylogenetics was used for the first time for *Namalycastis* to support population monophyly and recognition of the new species.The analysis, using both mitochondrial and nuclear data, corroborated the morphological analysis in suggesting that our specimens represent an as yet undescribed species, *Namalycastis jaya* **sp. n.**, which forms a monophyletic group among the sampled nereidid taxa. Finally, a taxonomic key for *Namalycastis* species recorded from the Indian region is provided.

## Introduction

Namanereidinae (Polychaeta: Nereididae) represents one of the most successful groups of colonizers of brackish waters (Wesenberg Lund 1958). The subfamily is currently recognized to consist of three genera: *Namalycastis* Hartman, 1959, *Namanereis* Chamberlin, 1919, and *Lycastoides* Johnson, 1903. *Namalycastis* is currently the most species-rich genus within Namanereidinae and it is also one of the most successful polychaetes in polluted coastal areas (Glasby 1999). The species are commonly found in littoral or supralittoral areas in association with decaying vegetation and other organic-rich areas on or close to the shore. The genus presents several adaptations to this low-salinity or semi-terrestrial habitat, including modifications to the eye, integument and epidermis (Sadasivan Tampi 1949, Storch and Welsch 1972), modification to the nephridia (Krishnan 1952, Florence Mary 1966), the production of large yolky eggs and an apparent tendency towards hermaphroditism or parthenogenesis (Glasby et al. 1990). Although taxonomic records of *Namalycastis* species from off the coast of the Indian subcontinent are not infrequent (Sinha and Das 1998, Glasby et al. 2003), the boundaries of the geographic distribution of the genus are poorly known. *Namalycastis indica* (Southern, 1921) represents both the easternmost (Chilika Lake, an inlet of the Bay of Bengal) and westernmost (Mumbai, Arabian Sea) records of the genus (Southern 1921, Mandal and Harkantra 2012). In addition, *Namalycastis abiuma* Müller in Grube, 1871 has been recorded as far south in India as the Kayankulam estuaries (Glasby 1999). As no obvious biogeographical barrier exists throughout the coastline of either the Arabian Sea or the Bay of Bengal, it is likely that the genus is more widespread in this area than indicated in the current record (see Glasby 1999).

Morphological variation between species of *Namalycastis* is often minute, presenting a possible problem for taxonomists. This is in part due to their simplified body form – lack of a notopodium and few types of chaetae – compared to other Nereididae. For example, *Namalycastis abiuma* was long considered a single, widespread species with a high level of intraspecific morphological variation, attributed to the differences in habitat choice. However, close investigations of the details of the serrations on the falciger blades of the species group revealed that American populations could be divided into at least two separate species, *Namalycastis abiuma sensu* Müller in Grube, 1871 and *Namalycastis borealis* Glasby, 1999. Nevertheless, Indo-Pacific populations of *Namalycastis abiuma* are still indistinguishable from their American counterparts. Because of the aforementioned, the inclusion of molecular data in association with phylogenetic analyses represents the first step in the molecular characterization of members of the genus and may assist with understanding the taxonomic boundaries within *Namalycastis*.

Here, we describe a new species of *Namalycastis* from the southwest coast of India on the basis of morphological investigations and corroborate the novelty of the species by phylogenetic analysis using both mitochondrial and nuclear loci. In addition, a morphological key to the different *Namalycastis* species recorded from India is provided.

### Material and methods

In March of 2009 and January of 2010, several polychaete samples were collected from the retting zone (upper intertidal zone characterized by accumulation of rotting coconut husks) of the Kadinamkulam estuary, near Thiruvananthapuram off the southwest coast of India. Specimens were sizeable enough to be collected by eye from rotting organic matter mixed with muddy sediments at the shoreline. The polychaetes associated with retting coconut husk were collected by breaking the coconut husk with hammer and chisel. Identifications were fascilitated by previous contributions and morphological keys (e.g., Glasby 1999). Approximately 20–40 segments of the posterior portion (without pygidium) were used for DNA extraction. Samples used for DNA work were fixed in 95% ethanol, whereas those for morphological investigation were relaxed in isotonic MgCl_2_, rapidly submerged in 95% ethanol (for proboscis everting), fixed in 10% formalin and later transferred to 70% ethanol. The fixed specimens then were dissected and mounted in polyvinyl lactophenol on microscope slides for permanent preservation. Dissections were carried out using an Olympus SZ61 stereomicroscope and the drawings were made with the help of an Olympus BX41 camera lucida. Images were captured using an AX10STAR Plus camera.

Total genomic DNA was extracted from the tissue samples following the extraction procedure of Miller et al. (1988) with minor modifications. Partial sequences of 16S ribosomal mtDNA, cytochrome *c* oxidase subunit I (COI) mtDNA and nuclear 18S rDNA were PCR-amplified using the following primers: 16SAR-L (5’-CGCCTGTTTATCAAAAACAT-3’) and 16SBRH (5’-CCGGTCTGAACTCAGATCACGT-3’) for 16S (Palumbi 1996); FR1d (5’ TTCTCCACCAACCACAARGAYATYGG -3’) and FR1d_t1 (5’- CACCTCAGGGTGTCCGAARAAYCARAA -3’) for COI (Ivanova et al. 2007); and 18SA (5’- AACCTGGTTGATCCTGCCAGT -3’) and 18SB (5’- TGATCCTTCCGCAGGTTCACCT -3’) for 18S (Medlin et al. 1988). The PCR used the following protocol: an initial 5 minute denaturation step at 94°C for all samples, followed by 30 seconds denaturation at 94°C (1 minute for 16S and 18S), 30 seconds annealing at 55°C (1 minute at 58°C for 16S and 18S), 2 minutes extension at 72°C (1 minute for 16S and 18S) and a final 5 minute extension step at 72°C for all samples; the program was run for 30 cycles (COI) or 35 cycles (16S and 18S). All PCR products were checked by gel electrophoresis on a 2% agarose gel and successful amplifications were purified using a PureFast® Genomic DNA purification kit (Helini Biomolecules, Chennai, India) following the instructions given by the manufacturer. Thereafter, cycle sequencing (using the same primers as mentioned above) and ethanol precipitation was carried out, and nucleotide sequencing was performed on an ABI 3500 XL Genetic Analyzer (Applied Biosystems, Foster City, CA).

Nucleotide sequences were deposited at NCBI (accession numbers HQ456363 and JN790065–67 for COI, HM138706 and JX483868–70 for 16S, and HQ157238 and JX483865- 67 for 18S) and type specimens were deposited in the collections of the WGRC- ZSI.

### DNA analyses

Sequence reconciliation of forward and reverse sequences was carried out using BioEdit ver. 7.0.5.2 (Hall 1999). The sequences then were aligned using MAFFT (Katoh et al. 2002) on the European Bioinformatics Institute website (http://www.ebi.ac.uk/Tools/msa/mafft/ ) applying default settings. A phylogenetic analysis was performed under the criterion of maximum parsimony in TNT (Goloboff et al. 2008). A heuristic search was performed using the New Technology search parameters, employing sectorial searching, with the tree fusing and ratcheting algorithms turned on. Trees were retrieved by a driven search using 100 initial addition sequences and requiring that the minimum length tree be found at least 5 times. All characters were equally weighted and non-additive, and gaps were treated as missing data. The results of the New Technology searches were subsequently resubmitted to TNT for TBR branch swapping. Support values for nodes also were estimated in TNT through standard bootstrap resampling, using 1000 iterations, each subjected to five iterations of ratcheting and three rounds of tree fusing after an initial five rounds of Wagner tree building. The trees were rooted at *Pectinaria koreni* (Malmgren, 1866) following Rousset et al. (2007).

**The following abbreviations are used in the text:**

PSU Practical Salinity Unit

NCBI National Center for Biotechnology Information

WGRC, ZSI Western Ghats Regional Centre, Zoological Survey of India.

ICZN International Code of Zoological Nomenclature

## Systematics

### Order Phyllodocida Dales, 1962. Family Nereididae Blainville, 1818. Subfamily Namanereidinae Hartman, 1959. Genus *Namalycastis* Hartman, 1959

#### Namalycastis jaya

Magesh, Kvist & Glasby, 2012 sp. n.

urn:lsid:zoobank.org:act:2C0921DC-72BA-4CCD-8DC6-E2D134621931

http://species-id.net/wiki/Namalycastis_jaya

Figures 2a–k , 3a–l

##### Type locality.

**Figure 1.**
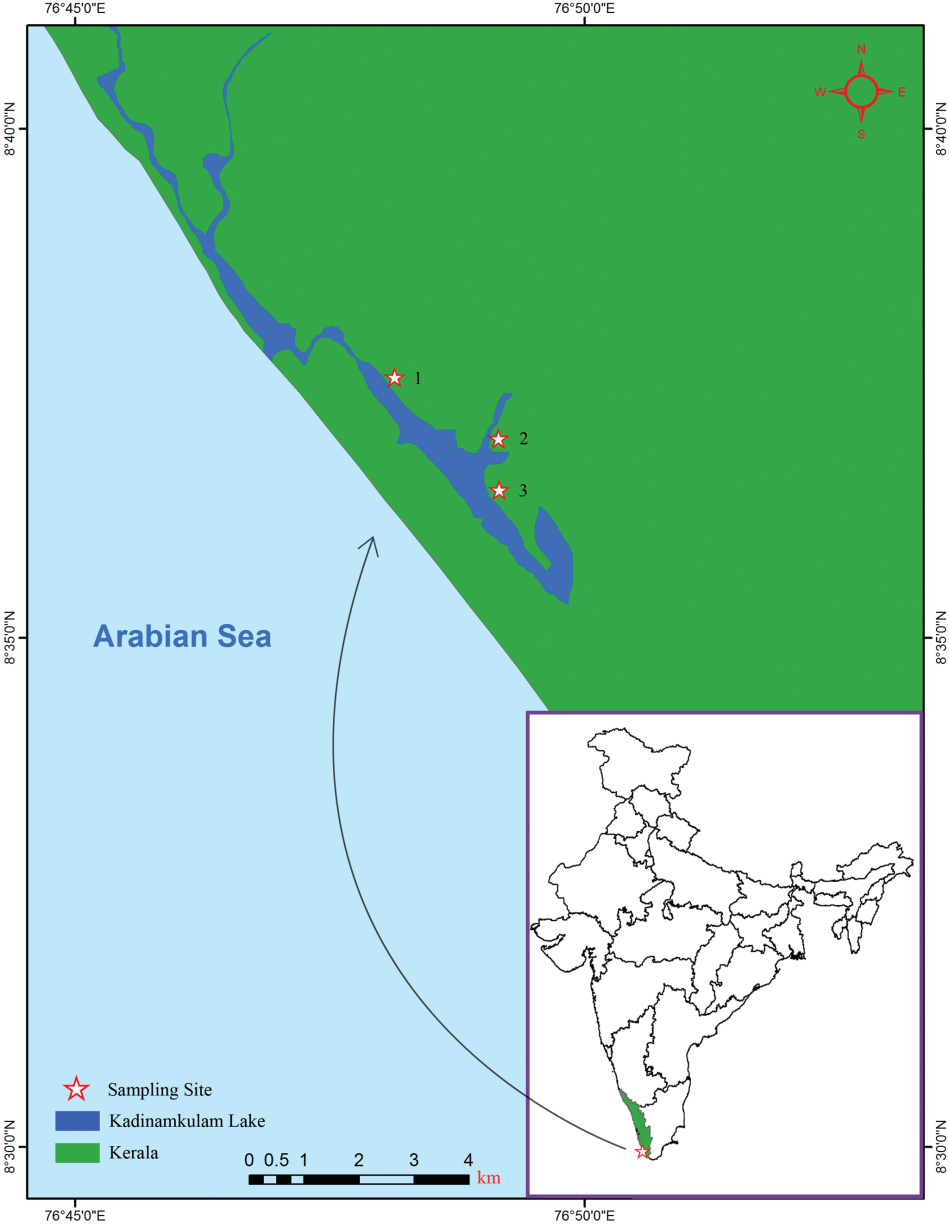
Map showing the positions of the collection localities of *Namalycastis jaya* sp. n. in Kerala , India. Site 1, Kadinamkulam estuary (8°37'33.34"N, 76°48'7.827"E); Site 2, Murukkumpuzha retting zone (8°36'57.473"N, 76°49'8.914"E); and Site 3, Kadinamkulam estuary (8°36'27.21"N, 76°49'9.474"E).

Murukkumpuzha retting zone, Thiruvananthapuram coast, Kerala, India, 8°36'57.47"N, 76°49'8.914"E (Fig. 1; site 2).

##### Type material.

Holotype AQJ1 (ZSI/WGRC/IR/IV 2330), adult specimen collected from muddy sediment in Murukkumpuzha retting zone, 8°36'57.47"N, 76°49'8.914"E (Fig. 1; site 2) by M. Magesh on 31 March, 2009. Paratypes, four specimens: AQJ2–4 (ZSI/WGRC/IR/IV 2331, 2332 and 2337) collected in Kadinamkulam estuary, Thiruvananthapuram coast, Kerala, India, 8°37'33.34"N, 76°48'7.827"E (Fig. 1; Site 1); and AQPE1 (ZSI/WGRC/IR/IV 2191), collected in muddy sediment from Kadinamkulam estuary, Thiruvananthapuram coast, Kerala, India, 8°36'27.21"N, 76°49'9.474"E (Fig. 1; site 3). All paratypes collected by M. Magesh on 21 January, 2010.

##### Description.

Holotype with body widest mid-anteriorly, tapering gradually anteriorly and posteriorly. Antennae small, distally subacute, aligned over mid-palps. Brown epidermal pigmentation present anterodorsally and posterodorsally.Prostomium triangular, deeply cleaved anteriorly. Longitudinal groove extending from tip to posterior part of prostomium,slightly indented laterally. Eyes 2 pairs, black, arranged obliquely, posterior pair considerably smaller. Posterodorsal tentacular cirri extending posteriorly to chaetiger 2. Jaws with 8 teeth, 4 subterminal and 4 ensheathed proximally.Acicular neuropodial ligule bilobed, superior lobe larger than inferior lobe. Dorsal cirri increasing in length posteriorly. Typically less than 4 sesquigomph spinigers in neuropodial supra-acicular fascicle in midbody. Notochaetae absent in all parapodia. Heterogomph chaetae with boss not lengthened. Supra-neuroacicular falcigers in chaetiger 10 with finely serrated blades, 9–12 teeth, approximately uniform in length. Sub-neuroacicular falcigers in chaetiger 10 with roughly serrated blades, about 20 teeth. Sub-neuroacicular spinigers in anterior region (up to segment 50) with blades finely serrated. Sub-neuroacicular spinigers in mid and posterior region (from about segment 70) with blades coarsely serrated proximally. Supra-neuroacicular spinigers in mid and posterior region with blades finely serrated. Pygidium with multi-incised rim, black with two lateral anal cirri.

**Figure 2.**
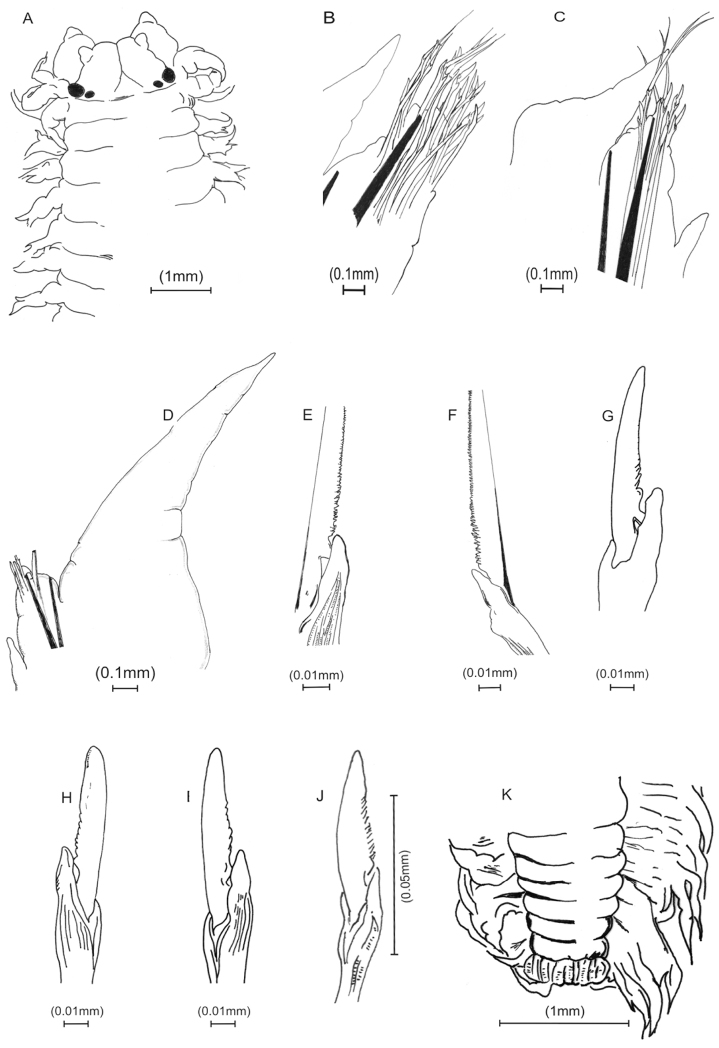
*Namalycastis jaya* sp. n.Holotype: **a** anterior end, dorsal view **b** anterior parapodium from chaetiger 8 **c** mid-body parapodium from chaetiger 80 **d** posterior parapodium from chaetiger 230 **e** sub-neuroacicular spiniger, chaetiger 10 **f** sub-neuroacicular spiniger, chaetiger 30 **g** sub-neuroacicular falciger, chaetiger 10 **h** sub-neuroacicular falciger, chaetiger 80 **i** supra-neuroacicular falciger, chaetiger 80 **j** supra-neuroacicular falciger, chaetiger 120 **k** pygidium, dorsal view.

**Figure 3.**
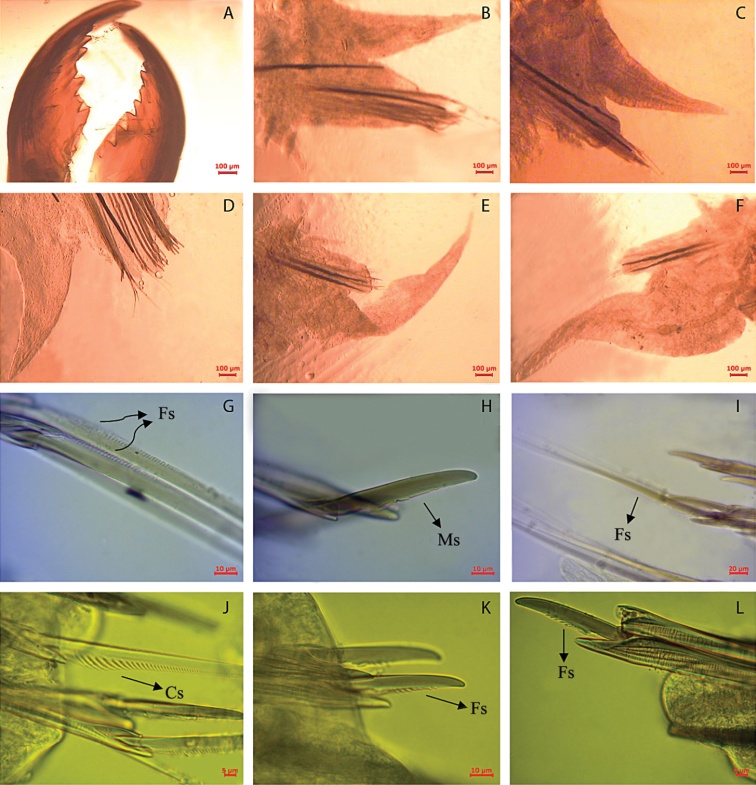
*Namalycastis jaya* sp. n.Holotype: **a** jaw pieces, ventromedial view **b** anterior parapodium from chaetiger 10 **c** parapodium from chaetiger 50 **d** parapodium from chaetiger 100 **e** posterior parapodium from chaetiger 210 **f** posterior parapodium from chaetiger 230 **g** sub-neuroacicular spiniger, chaetiger 10 **h** sub-neuroacicular falciger, chaetiger 109 **i** supra-neuroacicular spiniger, chaetiger 80 **j** sub-neuroacicular spiniger, chaetiger 210 **k** sub-neuroacicular falciger, chaetiger 210 **l** supra-neuroacicular falciger, chaetiger 20. **Fs** Finely serrated; **Cs** Coarsely serrated; **Ms** Medium serrated.

##### Etymology. 

Named in honour of Dr. Jayalalithaa Jayaram (born 1948), the current Chief Minister of Tamil Nadu State of India, in recognition of her contributions to the field of education (especially for impoverished people) and scientific research. The specific epithet is considered to be a noun in apposition.

##### Distribution.

Known only from the Thiruvananthapuram coast of southwest India (but see note below).

##### Taxonomic remarks.

*Namalycastis jaya* sp. n. resembles *Namalycastis elobeyensis* Glasby, 1999 and *Namalycastis hawaiiensis* Johnson, 1903 by virtue of lacking notochaetae. However, our new species differs from *Namalycastis elobeyensis* as the latterhas long antenna, equal size eyes, comparatively longer posterodorsal tentacular cirri, subconical acicular neuropodial ligule, tripartite pygidium and no epidermal pigmentation. *Namalycastis jaya* sp. n. also differs from *Namalycastis hawaiiensis*, by the latter possessing 35 to 70 teeth on the blades of the sub-neuroacicular falcigers in parapodia of chaetiger 10, mid-posterior falcigers with proximally coarsely serrated blades from chaetiger 120 (chaetiger 30 in smaller specimens) and by the absence of epidermal pigmentation. The lack of notochaetae sets *Namalycastis jaya* sp. n. apart from other Indian species, including *Namalycastis indica* Southern, 1921, the *Namalycastis abiuma* species group, *Namalycastis fauveli* Nageswara Rao, 1981 and the recently described species *Namalycastis glasbyi* Fernando and Rajasekaran, 2007. These other species typically have 1–3 notochaetae in at least some parapodia, except those in the anterior-most and posterior-most body. *Namalycastis jaya* sp. n. most closely resembles *Namalycastis abiuma*, but differs from the holotype of that species in having very short tentacular cirri (posterodorsal one only extending to chaetiger 2 as compared to chaetiger 5 in *Namalycastis abiuma*), in the very short, distally sub-acute antennae (antennae pointed and extending to end of palps in *Namalycastis abiuma*) and in lacking notochaetae (present from chaetiger 12 in *Namalycastis abiuma*). A key for taxonomic differentiation between species recorded from the Indian region is provided below.

Based on the above comparative account of the features used for identifying the species of the family Nereididae, the present species can be distinguished as a new species by the following combination of characters: (1) smooth and small antennae, (2) absence of notochaetae in all chaetigerous segments, (3) sub and supra-neuroacicular falcigers of parapodia 10 with finely serrated blades, (4) coarsely serrated teeth in sub-neuroacicular spinigers in mid-posterior region, (5) brown pygidium with multi incised rim and two lateral anal cirri, (6) jaws with 8 teeth, and (7) eye capsule protruded above the dorsal alignment of the head. In all of these features, the new species resembles *Namalycastis meraukensis* (*var. zeylancia*), described from Dondra, southern Sri Lanka by de Silva (1961). This taxon was considered to be a junior synonym of the *Namalycastis abiuma* species group by Glasby (1999). However, de Silva’s (1961) variety name is not an available name under the ICZN (1999; Article 15.2), and so the name *zeylanica* cannot technically be considered a synonym or elevated to a species to represent the present specimens. The name *Namalycastis jaya*sp. n. is therefore likely attributable to the Dondra population and the species more widely distributed throughout southern India and Sri Lanka. Interestingly, the Dondra specimens also live in the retting zone (C. Glasby, pers. obs. 1987).

##### Ecological note.

This species is able to sustain in polluted (sulphide rich and odorous) sediments and is especially associated with decaying materials such as bark and retting coir; the salinity at the collection localities ranged from 5–22 psu.

### Phylogeny

**Figure 4.**
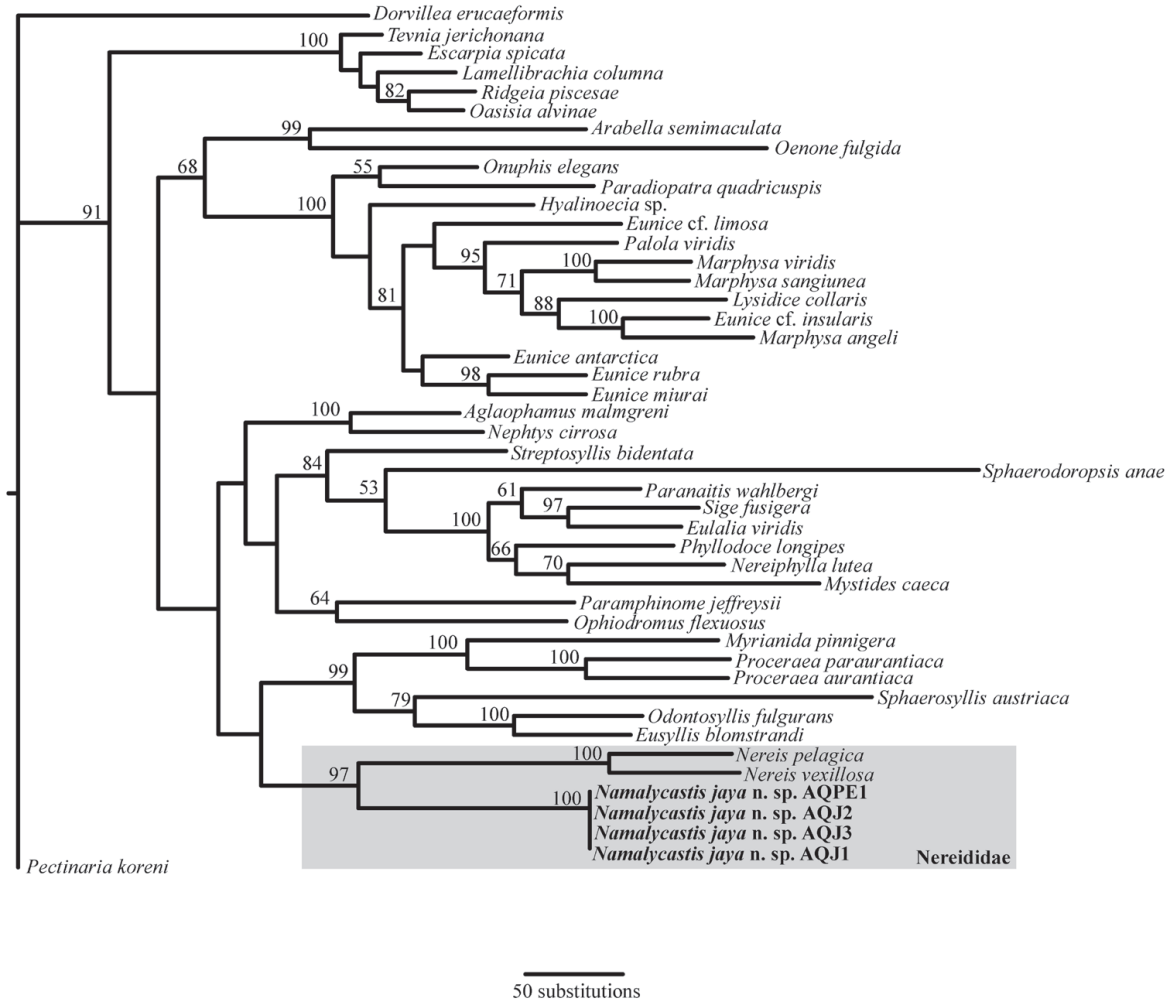
Strict consensus of five equally parsimonious trees from the TNT analysis (Length: 8426; CI: 0.357; RI: 0.543).Bootstrap support values are shown above the nodes and representatives of the new species are shown in bold font. See text for further discussion.

In order to rigorously test the familial placement of our species, the phylogenetic analysis used numerous specimens from various polychaete families as well as the only two members of Nereididae for which data were available for all of COI, 16S and 18S (*Nereis pelagica* Linnaeus, 1758and *Nereis vexillosa* Grube, 1849). The final molecular matrix contained 3789 aligned sites. The TNT analysis recovered 5 equally parsimonious trees, 8426 steps long and the strict consensus of these (Fig. 4) corroborates the morphological analysis in confirming the novelty of the species. The specimens of *Namalycastis jaya* sp. n.form a monophyletic group among the sampled nereidid taxa. This position is supported by a bootstrap value of 97% and the monophyly of the specimens received maximum support. The details of the remainder of the tree are presented in Fig. 4.

## Discussion

The monophyletic status of the mostly tropical or subtropical subfamily Namanereidinae has been confirmed by phylogenetic analyses (Fitzhugh 1987, Glasby 1991, 1999), yet the status of the genera within the subfamily have been debated. Hartman (1959) recognized three genera (*Namalycastis*, *Namanereis* and *Lycastoides*) as well as a monotypic fourth group of questionable generic status represented by *Lycastis geayi* Gravier, 1901. The doubt shed on the generic status of this group stems from uncertainty in the number of peristomial tentacles (Hartman 1959) and Glasby (1999) transferred *Lycastoides geayi* to *Namalycastis*, which enjoyed seniority over *Lycastis*. In addition, two monotypic genera within the subfamily, *Cryptonereis* Gibbs, 1971 and *Lycastilla* Solis-Weiss and Luis Espinasa, 1991, have since been synonymized by Glasby (1999). The three current genera in the subfamily share distinct synapomorphic features, including a combination of either reduced notopodia or the absence of dorsal cirrophores, as well as spherical palpostyles and an indistinct separation between neuropodia and notopodia (Glasby 1999). Much like *Namanereis* species, our new species lacks notochaetae in all parapodia but, like other species of *Namalycastis*, it does possess very short, conical antennae and posterior leaf-like dorsal cirri; features that define the genus.

*Namalycastis jaya* sp. n. represents the fifth species of *Namalycastis* recorded from India, the remaining species being the *Namalycastis abiuma* species group, *Namalycastis indica*, *Namalycastis fauveli* and *Namalycastis glasbyi*. We note that *Namalycastis glasbyi*, which is known only from the type locality, Mumbai, bears a close resemblance to *Namalycastis indica* (see Fernando and Rajasekaran 2007) for which verified records are known from the east coast of India, Sri Lanka and Bangladesh (Glasby 1999). Both species share the distinctive elongated dorsal cirri of posterior chaetigers, but completely lack associated molecular data. Together, these five species show a wide distribution across India and, at least the Indian members of the genus, seem to have a particular talent for inhabiting odd, inhospitable and vastly different surroundings. Species of the genus have been recorded from waters ranging dramatically in salinity, from drinkable freshwater to full salinity waters, freshwater container habitats such as plant-leaf axils (Glasby et al. 2003) and in severely contaminated waters such as those subjected to industrial pollution. This tolerance for varying environments may also indicate that the abundance of the genus is richer and its distribution wider than currently recognized.

### Key to the Indian species of genus *Namalycastis* Hartman, 1959

**Table:** 

1	Articulation of heterogomph chaetae with boss extraordinarily expanded; antennae minute	*Namalycastis fauveli*
–	Articulation of heterogomph chaetae with boss not extraordinarily expanded (equal or little longer); antennae extending beyond tip of prostomium	2
2	Notochaetae present in all or several parapodia; antennae distally pointed	3
–	Notochaetae absent in all parapodia; antennae distally sub-acute	*Namalycastis jaya* sp. n.
3	Anterior and posterior eyes more or less same size	4
–	Anterior eyes substantially smaller than posterior ones	*Namalycastis glasbyi*
4	Lengthy posterodorsal tentacular cirri reaching back to chaetiger 5–6 and tripartite pygidium	*Namalycastis indica*
–	Postero-dorsal tentacular cirri only reaching back to chaetiger 2–5; multi-incised pygidium	*Namalycastis abiuma* species group

## Supplementary Material

XML Treatment for Namalycastis jaya
